# Supplementary material for: MetaRibo-Seq measures translation in microbiomes
Source: Nat Commun. 2020 Jun 29;11:3268. doi: 10.1038/s41467-020-17081-z (PMC7324362; doi:10.1038/s41467-020-17081-z)
Supplement: Supplementary file 10 — Supplementary Data 7 [file 41467_2020_17081_MOESM10_ESM.zip › File2/Confidence_High_Taxonomy/177815_out.krona.html]

Javascript must be enabled to view this page.

members
magnitude
magnitudeUnassigned
count
unassigned
taxon
rank

177815\_out

400

2
400
superkingdom

976
400
phylum

200643
class
400

171549
order
400

815
399
family

399
816
genus

SRS014948\_contig\_number\_14408SRS016495\_contig\_number\_15110SRS017433\_contig\_number\_contig-100\_9616.9616SRS018427\_contig\_number\_8259SRS019030\_contig\_number\_contig-100\_1297.97191SRS019910\_contig\_number\_6663SRS019968\_contig\_number\_2815SRS022713\_contig\_number\_614SRS022713\_contig\_number\_1003SRS023583\_contig\_number\_2176SRS044535\_contig\_number\_1101SRS045004\_contig\_number\_2427SRS046369\_contig\_number\_8492SRS049402\_contig\_number\_9169SRS052027\_contig\_number\_14224SRS052078\_contig\_number\_215SRS056273\_contig\_number\_723SRS063489\_contig\_number\_6438SRS078665\_contig\_number\_7363SRS1041031\_contig\_number\_23437SRS1041037\_contig\_number\_14416SRS1041092\_contig\_number\_15283SRS143466\_contig\_number\_12123SRS143876\_contig\_number\_6865SRS144506\_contig\_number\_50049SRS145497\_contig\_number\_contig-100\_10334.10334SRS148159\_contig\_number\_22750SRS893256\_contig\_number\_contig-100\_660.24469SRS893380\_contig\_number\_contig-100\_757.26334
29


SRS043411\_contig\_number\_11621SRS048870\_contig\_number\_2533SRS058070\_contig\_number\_12248SRS075984\_contig\_number\_15385SRS077335\_contig\_number\_11SRS104975\_contig\_number\_3483SRS1055038\_contig\_number\_1450SRS142712\_contig\_number\_985
246787
species
8

671267

SRS1041132\_contig\_number\_5983SRS1041134\_contig\_number\_contig-100\_1590.75961SRS1041136\_contig\_number\_18846
species
3

1
species
2292944

SRS015369\_contig\_number\_5420

2
species

SRS023829\_contig\_number\_460SRS049712\_contig\_number\_22365
28116


SRS018351\_contig\_number\_9421
1897051
1
species


SRS013476\_contig\_number\_28185SRS014613\_contig\_number\_12237SRS014923\_contig\_number\_12586SRS015794\_contig\_number\_3006SRS045713\_contig\_number\_8923SRS064232\_contig\_number\_3721SRS064276\_contig\_number\_41036SRS075963\_contig\_number\_contig-100\_222.222SRS1041145\_contig\_number\_39772SRS1054691\_contig\_number\_6006SRS1055067\_contig\_number\_13767
2292935
species
11

291645

SRS016095\_contig\_number\_14355
1
species

457395

SRS013098\_contig\_number\_contig-100\_1272.287897SRS015890\_contig\_number\_2661SRS018984\_contig\_number\_10769SRS058770\_contig\_number\_contig-100\_1112.132280SRS075341\_contig\_number\_18458SRS077024\_contig\_number\_3435SRS1055034\_contig\_number\_9749SRS148511\_contig\_number\_17184SRS148874\_contig\_number\_5289SRS149879\_contig\_number\_21850SRS893230\_contig\_number\_11859
11
species

species
5
310297

SRS013382\_contig\_number\_527SRS015663\_contig\_number\_22809SRS047044\_contig\_number\_16257SRS050026\_contig\_number\_4609SRS148319\_contig\_number\_5700

2
species

SRS098717\_contig\_number\_15648SRS1041095\_contig\_number\_contig-100\_5396.29402
376806

28111

SRS048164\_contig\_number\_6930SRS1041032\_contig\_number\_5984
2
species


SRS011084\_contig\_number\_14020
1263056
species
1

species
2

SRS052697\_contig\_number\_32444SRS1055095\_contig\_number\_301
204516

357276

SRS013940\_contig\_number\_16763SRS018049\_contig\_number\_396SRS019286\_contig\_number\_2429SRS023715\_contig\_number\_2282SRS024549\_contig\_number\_468SRS024663\_contig\_number\_1133SRS043001\_contig\_number\_458SRS043768\_contig\_number\_24721SRS048981\_contig\_number\_3502SRS075021\_contig\_number\_1007SRS101268\_contig\_number\_393SRS1041038\_contig\_number\_16227SRS1041112\_contig\_number\_449SRS147139\_contig\_number\_26868
14
species


SRS050422\_contig\_number\_8609
457390
1
species

species
1

SRS020233\_contig\_number\_10722
28113

626931

SRS058723\_contig\_number\_1873
species
1

10
species

SRS013215\_contig\_number\_123SRS015854\_contig\_number\_contig-100\_208.120632SRS018575\_contig\_number\_5119SRS042284\_contig\_number\_995SRS045195\_contig\_number\_3922SRS057717\_contig\_number\_3108SRS1041090\_contig\_number\_174SRS143598\_contig\_number\_4927SRS147425\_contig\_number\_8007SRS893382\_contig\_number\_2450
818

species
1

SRS893358\_contig\_number\_2057
162156

46506

SRS013800\_contig\_number\_10840SRS014855\_contig\_number\_4786SRS016954\_contig\_number\_4304SRS017521\_contig\_number\_42980SRS020328\_contig\_number\_32001SRS020394\_contig\_number\_13632SRS021948\_contig\_number\_2599SRS022137\_contig\_number\_1921SRS049900\_contig\_number\_7747SRS053398\_contig\_number\_3451SRS055982\_contig\_number\_3824SRS056259\_contig\_number\_11715SRS057478\_contig\_number\_5042SRS063813\_contig\_number\_4898SRS076876\_contig\_number\_271SRS097920\_contig\_number\_21987SRS098571\_contig\_number\_2354SRS104400\_contig\_number\_contig-100\_2.307358SRS1055069\_contig\_number\_4149SRS1055076\_contig\_number\_9210SRS140492\_contig\_number\_2812SRS142890\_contig\_number\_8489SRS147377\_contig\_number\_5366SRS147614\_contig\_number\_1443SRS149181\_contig\_number\_24742
species
25


SRS024435\_contig\_number\_4876SRS049959\_contig\_number\_16259SRS098655\_contig\_number\_8349SRS149325\_contig\_number\_10181
291644
species
4

2
species
2292943

SRS056519\_contig\_number\_1073SRS140645\_contig\_number\_383

820

SRS017821\_contig\_number\_21133SRS021219\_contig\_number\_3377SRS023971\_contig\_number\_1255SRS046717\_contig\_number\_7710SRS050752\_contig\_number\_12700SRS076976\_contig\_number\_1093SRS077849\_contig\_number\_4339SRS149784\_contig\_number\_17531SRS971275\_contig\_number\_20390
species
9

4
species
329854

SRS015960\_contig\_number\_15271SRS018623\_contig\_number\_13684SRS018836\_contig\_number\_16388SRS893369\_contig\_number\_10201


SRS017433\_contig\_number\_contig-100\_1777.71133SRS104912\_contig\_number\_4304SRS1055049\_contig\_number\_2734
371601
species
3

1262742

SRS893300\_contig\_number\_188
1
species

1
species
1638780

SRS055966\_contig\_number\_63

821

SRS011134\_contig\_number\_24982SRS011239\_contig\_number\_13152SRS011302\_contig\_number\_4835SRS011405\_contig\_number\_8141SRS012849\_contig\_number\_20285SRS012902\_contig\_number\_73SRS013158\_contig\_number\_2595SRS013521\_contig\_number\_13618SRS013687\_contig\_number\_contig-100\_1425.173006SRS013965\_contig\_number\_37621SRS014235\_contig\_number\_39389SRS014287\_contig\_number\_15829SRS014412\_contig\_number\_14SRS014534\_contig\_number\_258SRS014683\_contig\_number\_12376SRS014736\_contig\_number\_8126SRS014979\_contig\_number\_35132SRS015065\_contig\_number\_9661SRS015190\_contig\_number\_10921SRS015217\_contig\_number\_26808SRS015431\_contig\_number\_70174SRS015578\_contig\_number\_8951SRS015579\_contig\_number\_26134SRS015694\_contig\_number\_15979SRS015782\_contig\_number\_16293SRS016056\_contig\_number\_3339SRS016132\_contig\_number\_1219SRS016203\_contig\_number\_9562SRS016335\_contig\_number\_279SRS016381\_contig\_number\_2703SRS016629\_contig\_number\_1843SRS016753\_contig\_number\_contig-100\_1224.56514SRS016897\_contig\_number\_200SRS017103\_contig\_number\_26094SRS017247\_contig\_number\_contig-100\_1249.62553SRS017307\_contig\_number\_16279SRS017375\_contig\_number\_441SRS017745\_contig\_number\_891SRS017859\_contig\_number\_contig-100\_1346.50457SRS018888\_contig\_number\_9177SRS018936\_contig\_number\_849SRS019068\_contig\_number\_42075SRS019161\_contig\_number\_14352SRS019178\_contig\_number\_3959SRS019267\_contig\_number\_1834SRS019381\_contig\_number\_12955SRS019397\_contig\_number\_7960SRS019445\_contig\_number\_18266SRS019496\_contig\_number\_3571SRS019601\_contig\_number\_20353SRS019808\_contig\_number\_19608SRS020508\_contig\_number\_885SRS020622\_contig\_number\_6430SRS021153\_contig\_number\_1044SRS021484\_contig\_number\_12289SRS022524\_contig\_number\_14425SRS023914\_contig\_number\_2031SRS024009\_contig\_number\_326SRS024075\_contig\_number\_9119SRS024492\_contig\_number\_3049SRS024625\_contig\_number\_9848SRS042966\_contig\_number\_14190SRS043667\_contig\_number\_contig-100\_1372.36901SRS045244\_contig\_number\_661SRS045645\_contig\_number\_131SRS045739\_contig\_number\_contig-100\_1243.66314SRS046068\_contig\_number\_755SRS046502\_contig\_number\_10738SRS046810\_contig\_number\_contig-100\_38.39SRS047014\_contig\_number\_29312SRS047433\_contig\_number\_10727SRS047433\_contig\_number\_23148SRS049446\_contig\_number\_9542SRS049995\_contig\_number\_35246SRS050520\_contig\_number\_149SRS050801\_contig\_number\_contig-100\_676.28723SRS050925\_contig\_number\_26052SRS050941\_contig\_number\_contig-100\_1301.115632SRS050998\_contig\_number\_16957SRS051031\_contig\_number\_contig-100\_2043.174521SRS051610\_contig\_number\_8677SRS051882\_contig\_number\_12184SRS052576\_contig\_number\_contig-100\_6.15515SRS053087\_contig\_number\_2606SRS053356\_contig\_number\_45924SRS054059\_contig\_number\_1623SRS054352\_contig\_number\_15930SRS054590\_contig\_number\_793SRS054905\_contig\_number\_20481SRS056537\_contig\_number\_41656SRS057480\_contig\_number\_10345SRS058268\_contig\_number\_34SRS062654\_contig\_number\_14859SRS062701\_contig\_number\_6749SRS063127\_contig\_number\_2562SRS063190\_contig\_number\_11372SRS063370\_contig\_number\_8610SRS063518\_contig\_number\_16279SRS063928\_contig\_number\_1174SRS063985\_contig\_number\_26798SRS064645\_contig\_number\_1321SRS064757\_contig\_number\_23367SRS064973\_contig\_number\_368SRS065176\_contig\_number\_contig-100\_1593.95987SRS065397\_contig\_number\_983SRS065504\_contig\_number\_43SRS074586\_contig\_number\_4677SRS074964\_contig\_number\_3610SRS075078\_contig\_number\_19119SRS075716\_contig\_number\_3954SRS075773\_contig\_number\_15213SRS075821\_contig\_number\_6598SRS075878\_contig\_number\_891SRS076756\_contig\_number\_1276SRS077194\_contig\_number\_18098SRS077231\_contig\_number\_23071SRS077294\_contig\_number\_637SRS077392\_contig\_number\_36669SRS077454\_contig\_number\_9580SRS077502\_contig\_number\_13375SRS077552\_contig\_number\_7147SRS077641\_contig\_number\_15410SRS078176\_contig\_number\_17326SRS078242\_contig\_number\_30956SRS078419\_contig\_number\_18544SRS097889\_contig\_number\_18153SRS097958\_contig\_number\_2565SRS098061\_contig\_number\_6021SRS098073\_contig\_number\_16936SRS098644\_contig\_number\_4230SRS098827\_contig\_number\_1642SRS098881\_contig\_number\_4224SRS100021\_contig\_number\_5786SRS100907\_contig\_number\_7882SRS101376\_contig\_number\_13950SRS101433\_contig\_number\_11207SRS103987\_contig\_number\_3328SRS104084\_contig\_number\_12958SRS1041036\_contig\_number\_2360SRS1041037\_contig\_number\_10198SRS1041039\_contig\_number\_21562SRS1041090\_contig\_number\_contig-100\_562.45263SRS1041091\_contig\_number\_18237SRS1041116\_contig\_number\_3817SRS1041118\_contig\_number\_3044SRS1041130\_contig\_number\_5203SRS1041137\_contig\_number\_22862SRS1041138\_contig\_number\_11140SRS1041140\_contig\_number\_22339SRS1041141\_contig\_number\_8008SRS1041143\_contig\_number\_contig-100\_1406.44411SRS1041144\_contig\_number\_6251SRS1041157\_contig\_number\_contig-100\_3168.37539SRS1041164\_contig\_number\_636SRS104327\_contig\_number\_1568SRS104485\_contig\_number\_200SRS105082\_contig\_number\_11108SRS105153\_contig\_number\_24366SRS1054716\_contig\_number\_9171SRS1054928\_contig\_number\_835SRS1055022\_contig\_number\_14236SRS1055056\_contig\_number\_contig-100\_71.77271SRS1055083\_contig\_number\_8346SRS140513\_contig\_number\_10283SRS142503\_contig\_number\_30874SRS142542\_contig\_number\_contig-100\_1435.149158SRS142599\_contig\_number\_13687SRS142781\_contig\_number\_2480SRS142923\_contig\_number\_6804SRS143070\_contig\_number\_1411SRS143085\_contig\_number\_3986SRS143148\_contig\_number\_14706SRS143181\_contig\_number\_5501SRS143342\_contig\_number\_11646SRS143372\_contig\_number\_6521SRS143417\_contig\_number\_30939SRS143722\_contig\_number\_5009SRS143895\_contig\_number\_36755SRS143991\_contig\_number\_27065SRS144135\_contig\_number\_27579SRS144183\_contig\_number\_24166SRS144297\_contig\_number\_9578SRS144362\_contig\_number\_464SRS144537\_contig\_number\_48174SRS144714\_contig\_number\_29215SRS144753\_contig\_number\_12074SRS145308\_contig\_number\_2852SRS145497\_contig\_number\_11130SRS146764\_contig\_number\_17637SRS146813\_contig\_number\_16025SRS147022\_contig\_number\_1211SRS147271\_contig\_number\_12355SRS147346\_contig\_number\_9491SRS147653\_contig\_number\_4866SRS147766\_contig\_number\_2342SRS147977\_contig\_number\_8353SRS148091\_contig\_number\_5641SRS148196\_contig\_number\_37566SRS148253\_contig\_number\_423SRS148424\_contig\_number\_26537SRS148721\_contig\_number\_11558SRS148817\_contig\_number\_8140SRS148970\_contig\_number\_214SRS149075\_contig\_number\_11679SRS149244\_contig\_number\_1803SRS150029\_contig\_number\_contig-100\_963.122112SRS892882\_contig\_number\_16150SRS893170\_contig\_number\_1194SRS893172\_contig\_number\_14952SRS893252\_contig\_number\_19540SRS893253\_contig\_number\_506SRS893259\_contig\_number\_5211SRS893279\_contig\_number\_11644SRS893288\_contig\_number\_3084SRS893292\_contig\_number\_6623SRS893295\_contig\_number\_10207SRS893300\_contig\_number\_8172SRS893327\_contig\_number\_7426SRS893342\_contig\_number\_7759SRS893366\_contig\_number\_20908SRS893373\_contig\_number\_20280
species
221


SRS012273\_contig\_number\_30723SRS012969\_contig\_number\_15424SRS014313\_contig\_number\_4188SRS018133\_contig\_number\_2540SRS022071\_contig\_number\_32098SRS043701\_contig\_number\_6759SRS048060\_contig\_number\_1447SRS049773\_contig\_number\_21632SRS050299\_contig\_number\_1329SRS062427\_contig\_number\_4225SRS143523\_contig\_number\_8607
47678
species
11

1
species

SRS055017\_contig\_number\_26644
310298

6
species
817

SRS018541\_contig\_number\_10399SRS023346\_contig\_number\_3948SRS077589\_contig\_number\_1710SRS104636\_contig\_number\_contig-100\_233.142273SRS1055043\_contig\_number\_22323SRS1055099\_contig\_number\_5370

674529

SRS016989\_contig\_number\_66
1
species


SRS104693\_contig\_number\_5093
469585
1
species

1263042

SRS077127\_contig\_number\_3364
species
1


SRS148784\_contig\_number\_3862
1262743
species
1

family
1
2005525

genus
1
375288

species
1
387661

SRS019693\_contig\_number\_2977
